# Residual stenosis after carotid artery stenting: Effect on periprocedural and long-term outcomes

**DOI:** 10.1371/journal.pone.0216592

**Published:** 2019-09-09

**Authors:** Jihoon Kang, Jeong-Ho Hong, Beom Joon Kim, Hee-Joon Bae, O-Ki Kwon, Chang Wan Oh, Cheolkyu Jung, Ji Sung Lee, Moon-Ku Han

**Affiliations:** 1 Department of Neurology, Seoul National University Bundang Hospital, Seoul National University, Seongnam, Republic of Korea; 2 Department of Neurology, Dongsan Medical Center, Keimyung University, Daegu, Republic of Korea; 3 Department of Neurosurgery, Seoul National University Bundang Hospital, Seoul National University, Seongnam, Republic of Korea; 4 Department of Radiology, Seoul National University Bundang Hospital, Seoul National University, Seongnam, Republic of Korea; 5 Clinical Research Center, Asan Medical Center, Ulsan University, Seoul, Republic of Korea; Medical University Innsbruck, AUSTRIA

## Abstract

**Objective:**

This study investigated the effect of residual stenosis after carotid artery stenting (CAS) on periprocedural and long-term outcomes.

**Methods:**

Patients treated with CAS for symptomatic or asymptomatic carotid arterial stenosis were consecutively enrolled. Residual stenosis was estimated from post-procedure angiography findings. The effects of residual stenosis on 30-day periprocedural outcome and times to restenosis and clinical outcome were analyzed using logistic regression models and Wei-Lin-Weissfeld models, respectively.

**Results:**

A total of 412 patients (age, 64.7 ± 17.0 years; male, 82.0%) were enrolled. The median baseline stenosis was 80% (interquartile range [IQR], 70–90%), which improved to 10% (0–30%) for residual stenosis. Residual stenosis was significantly associated with periprocedural outcome (adjusted odds ratio, 0.983; 95% confidence interval [CI], 0.965–0.999, P = 0.01) after adjustment for baseline stenosis, age, hypertension, symptomaticity, and statin use. Over the 5-year observation period, residual stenosis did not increase the global hazard for restenosis and clinical outcome (adjusted hazard ratio, 1.011; 95% CI, 0.997–1.025. In the event-specific model, residual stenosis increased the hazard for restenosis (adjusted hazard ratio, 1.041; 1.012–1.072) but not for clinical outcome (adjusted hazard ratio, 1.011; 0.997–1.025).

**Conclusions:**

Residual stenosis after carotid artery stenting may be useful to predict periprocedural outcome and restenosis.

## Introduction

Carotid artery stenting (CAS) has become the first line therapeutic option for managing patients with significant carotid stenosis.[1–3] As like the previous standard and effective therapeutic strategy of carotid endarterectomy (CEA), CAS has been shown to decrease the long-term cardiovascular risk to approximately 1% per year.[1,2]

A major concern associated with CAS is its 3–7% rate of periprocedural adverse outcomes, which is generally higher than that of CEA.[4] Since the stenting procedure itself is associated with various risks, such as plaque disruption with distal embolization, perfusion injury, and systemic hemodynamic instability,[5] it may result in detrimental ischemic or hemorrhagic stroke or cardiac events.[6–8] Although the refinement of procedural techniques and use of intensive monitoring system have contributed to decreasing such adverse outcomes,[9–11] large clinical trials have still demonstrated substantial rates of periprocedural outcomes.[12,13]

The immediate procedural outcome is determined based on underlying atheroma,[14] stent and angioplasty techniques.[15,16] As these factors are related to post-CAS outcome,[17] it has been proposed that residual stenosis after procedure can act as a surrogate biomarker for predicting periprocedural outcomes. Empirically, a target of less than 30% residual stenosis has been recommended[18,19]; however, a lesser degree of improvement might be sufficient to restore the perfusion of the cerebral hemisphere and decrease the plaque friability.[20,21] Nonetheless, the question remains as to whether high residual stenosis increases rates of restenosis and long-term adverse clinical outcome.[22]

To address these questions, this study aimed to investigate whether residual stenosis after CAS was associated with peri-procedural and long-term adverse outcome of restenosis and clinical events.

## Methods

### Standard protocol approvals, registrations, and patient consents

The local institutional review board of Seoul National University Bundang Hospital (SNUBH) approved the study protocol with a waiver of informed consent due to the retrospective registry-based study design and minimal risk to participants.

### Study subjects and data collection

This study identified patient treated with CAS due to symptomatic and asymptomatic carotid artery stenosis at SNUBH, Republic of Korea using the institutional registry and electronic medical health records (EHR). Of them, this study consecutively included who treated between July 2003 and April 2013.[23,24] Demographic characteristics, risk factors, such as hypertension, diabetes mellitus, dyslipidemia, and current smoking, and clinical information during the hospital stay were obtained by reviewing the registry database and EHR. Procedure-related hemodynamic instability, asymptomatic dissection, and embolization within 24 hours were recorded.[25]

After discharge, patients were advised to visit the outpatient clinic regularly for routine check-ups and the emergency room in the event that they suspected any new neurologic symptom. During follow-up, information concerning stroke, myocardial infarction (MI), and death was by reviewing the patient’s medical records. Stroke was defined as acute-onset focal neurologic deficit with compatible lesions of both ischemic and hemorrhagic stroke on imaging study. MI denoted that cardiologists confirmed acute coronary syndrome with supportive laboratory finding. Restenosis was defined as ipsilateral revascularization or the detection of 70–99% stenosis or occlusion on follow-up examination when were generally performed at first 3 months and then annually and was suspicious of stroke.

Periprocedural outcome was defined as a procedure-related event of hemodynamic instability, asymptomatic dissection, or asymptomatic embolization and a 30-day clinical outcome of stroke, myocardial infarction, and any cause of death. Hemodynamic instability denoted the hypotension (systolic blood pressure ≤ 80 mmHg) or bradycardia (heart rate ≤ 50 per minute).[23,26] Asymptomatic embolization was the of magnetic resonance (MR)—diffusion weighted imaging confirmed the cerebral infarction without any neurologic symptom. When the cerebral arterial dissection appearance was definitely observed in post-procedural digital subtraction angiography, computed tomography angiography or MR angiography without clinical neurologic symptom, it was operationally adjudicated as asymptomatic dissection. Long-term outcome was defined as the time from the procedure to restenosis or composite of stroke, MI, and any cause of death.

### CAS protocol

The degree of carotid artery stenosis was estimated using the method of the North American Symptomatic Carotid Endarterectomy Trial study.[27] Physicians made a final decision to perform CAS considering the degree and characteristics of baseline stenosis, presence or absence of symptomatic vessels, and individual patient’s medical and stroke profiles.[28] If carotid stenosis caused transient ischemic stroke, transient monocular blindness, or any ischemic stroke in the corresponding arterial territory within 180 days before the procedure, it was considered symptomatic.

Subjects were administered aspirin and clopidogrel before at least 7 days of elective procedure and occasionally with a loading dose of aspirin (300mg) and clopidogrel (300 mg) in emergent situations. The technical details of the procedure have been previously reported.[15,29] Briefly, patients received intravenous heparin infusion at the start of the procedure and intermittently thereafter. After successful placement of the guiding catheter, distal embolic protective devices and/or proximal ballooning before stent deployment were employed. Pre- and post-stent ballooning were performed empirically according to the decision of the interventionist.

### Statistical analysis

The baseline characteristics were compared between dichotomized residual stenosis groups (<20% versus ≥20%). In bivariate analysis, periprocedural outcome and time to restenosis and clinical outcome were analyzed according to dichotomized residual stenosis status using the Pearson χ^2^ test and log-rank test.

The non-linearity of the relation between continuous residual stenosis and periprocedural outcome and long-term outcomes were evaluated using the restricted cubic spline method with multiple logistic regression and Cox proportional hazard models, respectively. In the multiple logistic model, the probability of periprocedural outcome according to the degree of residual stenosis was calculated. To assess long-term outcomes, the Wei-Lin-Weissfeld (WLW) model was used to calculate the global and event-specific hazard ratios of degree of residual stenosis for restenosis and clinical outcome. As the sensitivity analysis, Cox proportional hazard models were constructed for composite of restenosis and clinical outcome and each outcome. The model fits of WLW model and Cox proportional hazard models were also estimated. The SPSS software (version 21.0, IBM, USA) and R program (version 3.3.3, R Foundation) were used for statistical analyses.

## Results

### Baseline characteristics

Four hundred twelve subjects were consecutively enrolled. Their mean age was 64.7 ± 17.0 years, and 82.0% were male. Symptomatic carotid stenosis was present in 55.3% of cases. The median degree of baseline carotid stenosis was 80% (interquartile range (IQR), 70–90%), with similar distribution in both symptomatic and asymptomatic stenosis cases.

The median residual stenosis after CAS was 10% (IQR, 0–30%) (S1 Fig), and subjects were divided into high (43.2%) and low residual stenosis groups according to its distribution (≥20% versus <20%). Among the baseline characteristics, the high residual stenosis was significantly associated with elderly, higher proportion of basal stenosis, hypertension and statin use (Table 1).

**Table 1:** Comparisons of baseline characteristics according to residual stenosis status.

Variables	Low residual stenosis (n = 234)	High residual stenosis (n = 178)	P value
Male	193 (82.5%)	145 (81.5%)	0.79
Age, y, mean ± SD	67.8 ± 10.8	70.2 ± 8.7	0.01*
Baseline stenosis, median (IQR)	80 (70–90)	80 (70–90)	0.02**
Baseline stenosis, mean ± SD	77.7 ± 12.5	80.9 ± 9.1	0.02**
Symptomatic (n = 228)	80 (70–90)	80 (70–90)	0.06**
Asymptomatic (n = 184)	80 (70–90)	80 (70–90)	0.17**
Contralateral stenosis	62 (26.5%)	43 (24.2%)	0.59
Hypertension	165 (70.5%)	140 (78.7%)	0.06
Diabetes mellitus	90 (38.5%)	63 (35.4%)	0.52
Dyslipidemia	83 (35.5%)	74 (41.6%)	0.21
Current smoker	90 (38.5%)	57 (32.0%)	0.18
Antiplatelet treatment	231 (98.7%)	178 (100.0%)	0.13
Aspirin	228 (97.4%)	177 (99.4%)	0.12
Clopidogrel	210 (89.7%)	167 (93.8%)	0.14
Statin use	116 (49.6%)	114 (64.0%)	0.003

Values are presented as number of patients (percentage) unless otherwise indicated. P values were obtained using the Pearson χ^2^ test, t-test (*), or Mann-Whitney U test (**). IQR, interquartile range.

### Periprocedural outcome and residual stenosis

Periprocedural outcome occurred in 79 subjects, of which 66 were procedure-related and 14 were clinical outcomes (Table 2). The rates of periprocedural outcome significantly differed between the high and low residual stenosis groups (14.0% and 23.5%, P = 0.02).

**Table 2:** Periprocedural outcome and residual stenosis.

Outcomes	All subjects	Low residual stenosis (n = 234)	High residual stenosis (n = 178)	P value
Procedure-related outcome	66 (16.0%)	46 (19.7%)	20 (11.2%)	0.02
Hemodynamic instability	42 (10.2%)	25 (10.7%)	17 (9.6%)	0.75
Dissection	16 (3.9%)	14 (6.0%)	2 (1.1%)	0.02
Embolization, asymptomatic	8 (1.9%)	7 (3.0%)	1 (0.6%)	0.15
Clinical outcome	14 (3.4%)	9 (3.8%)	5 (2.8%)	0.79
Ischemic stroke, non-fatal	4 (1.0%)	3 (1.3%)	1 (0.6%)	0.46
Hemorrhagic stroke, non-fatal	3 (0.7%)	1 (0.4%)	2 (1.1%)	0.54
Myocardial infarction, non-fatal	1 (0.2%)	1 (0.4%)	0 (0.0%)	0.38
Death other than cardiovascular disease event	5 (1.2%)	4 (1.7%)	2 (1.1%)	0.64
Hemorrhagic stroke, fatal	1 (0.2%)	1 (0.4%)	0 (0.0%)	0.38
Periprocedural outcome (procedure-related or clinical outcome)	80 (19.4%)	55 (23.5%)	25 (14.0%)	0.02

Procedure-related outcome denotes any asymptomatic event of hemodynamic instability (hypotension and/or bradycardia), dissection, and embolization occurring within 24 hours of the procedure. Clinical outcome was the composite of non-fatal stroke (ischemic and hemorrhagic), non-fatal myocardial infarction, and fatal stroke (ischemic and hemorrhagic) and death other than cardiovascular event within 1 month. P values were calculated using the Pearson χ^2^ test or Fisher’s exact test, as appropriate.

**Fig 1:**
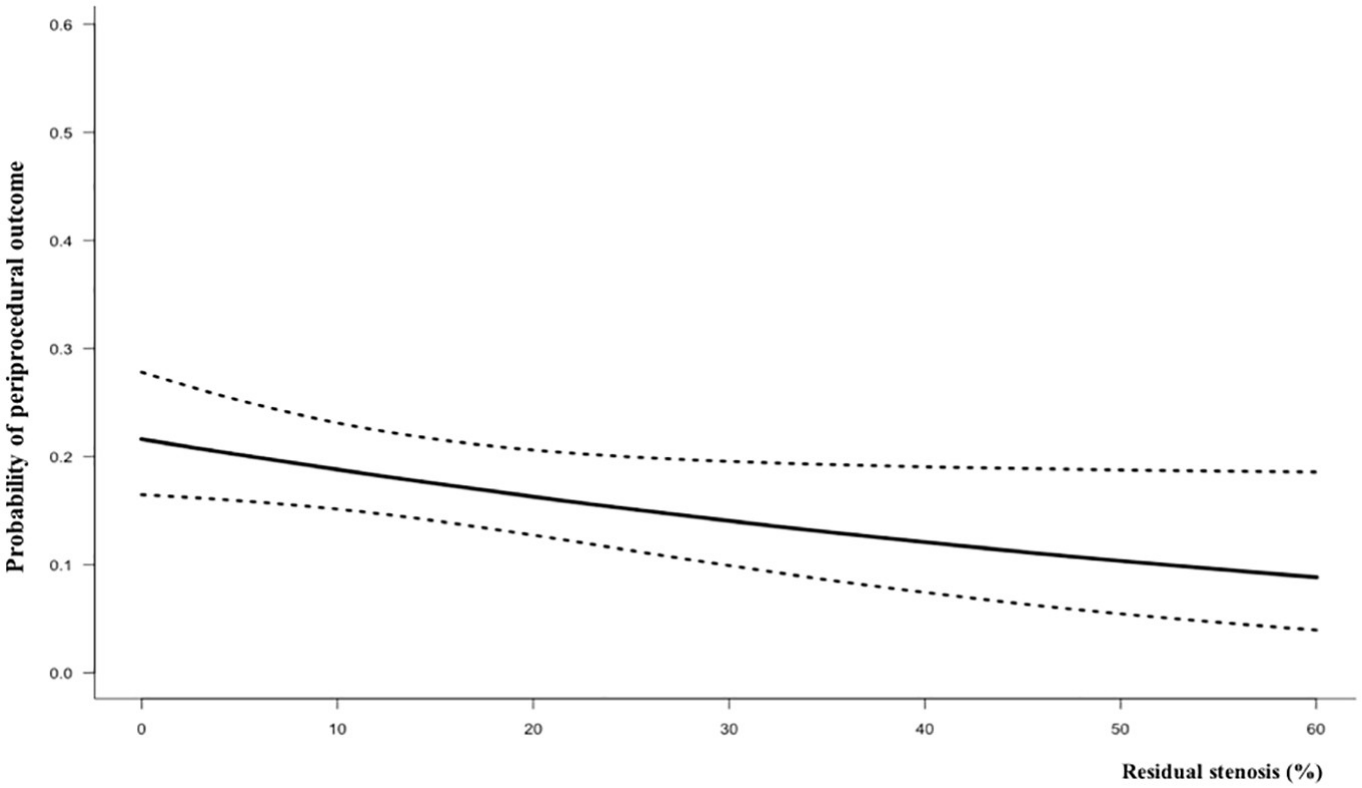
Probability of periprocedural outcome according to the degree of residual stenosis.

The multivariate logistic regression model with restricted cubic spline technique indicated that residual stenosis was not fitted to a non-linear trend with periprocedural outcome (P for non-linearity = 0.89). Residual stenosis demonstrated an independent association with the periprocedural outcome (adjusted odds ratio, 0.983; 95% confidence interval, 0.965–0.999) after adjustment for age, hypertension, baseline stenosis, symptomaticity, and statin use (S1 Table). The probability of periprocedural outcome showed an inverse relationship with residual stenosis (Fig 1).

### Long-term outcome and residual stenosis

**Fig 2:**
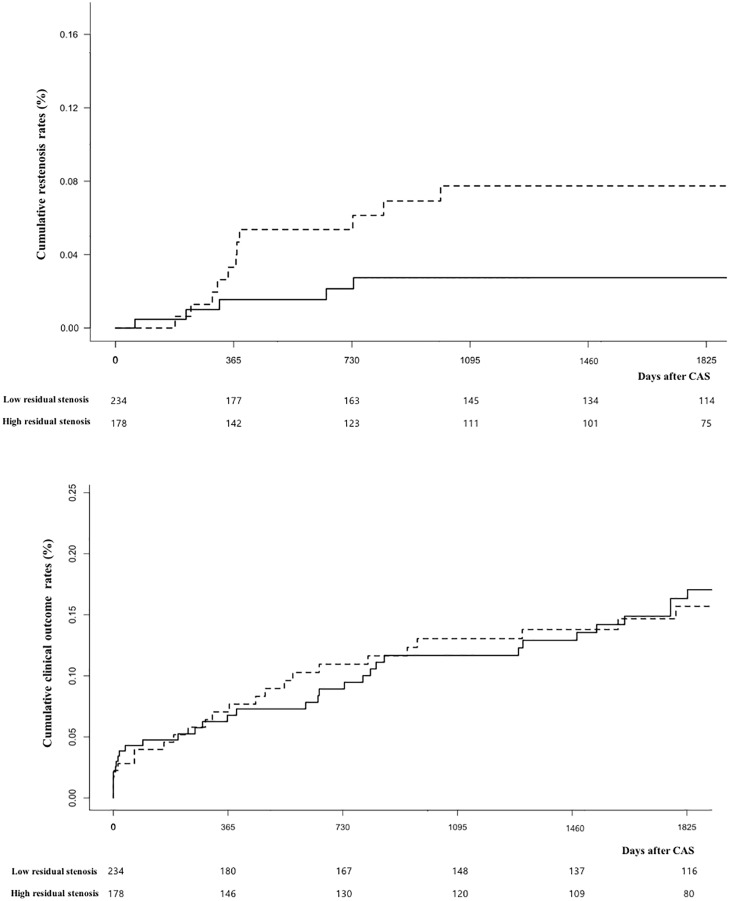
Kaplan-Meier survival curves of residual stenosis for hazards of restenosis (upper) and clinical outcome (lower). The P values determined by log-rank test were 0.047 (upper) and 0.86 (lower), respectively. The solid line indicates low residual stenosis, and the dotted line indicates high residual stenosis.

Subjects were followed for a median of 1817 days (IQR, 820–2686 days). The cumulative incidence rates of restenosis were estimated at 2.3% at 1 year, 3.6% at 2 years, 5.0% at 3 years, and 5.0% at 5 years. The incidence rates of clinical outcome at these time points were estimated at 6.9%, 9.9%, 12.3%, and 16.1%, respectively. Cumulative restenosis rates significantly differed between the high and low residual stenosis groups (7.7% vs. 2.3%, P = 0.047). The rates of clinical outcome did not significantly differ between the high and low residual stenosis groups (15.7% vs. 16.3%, P = 0.86) (Fig 2).

**Fig 3:**
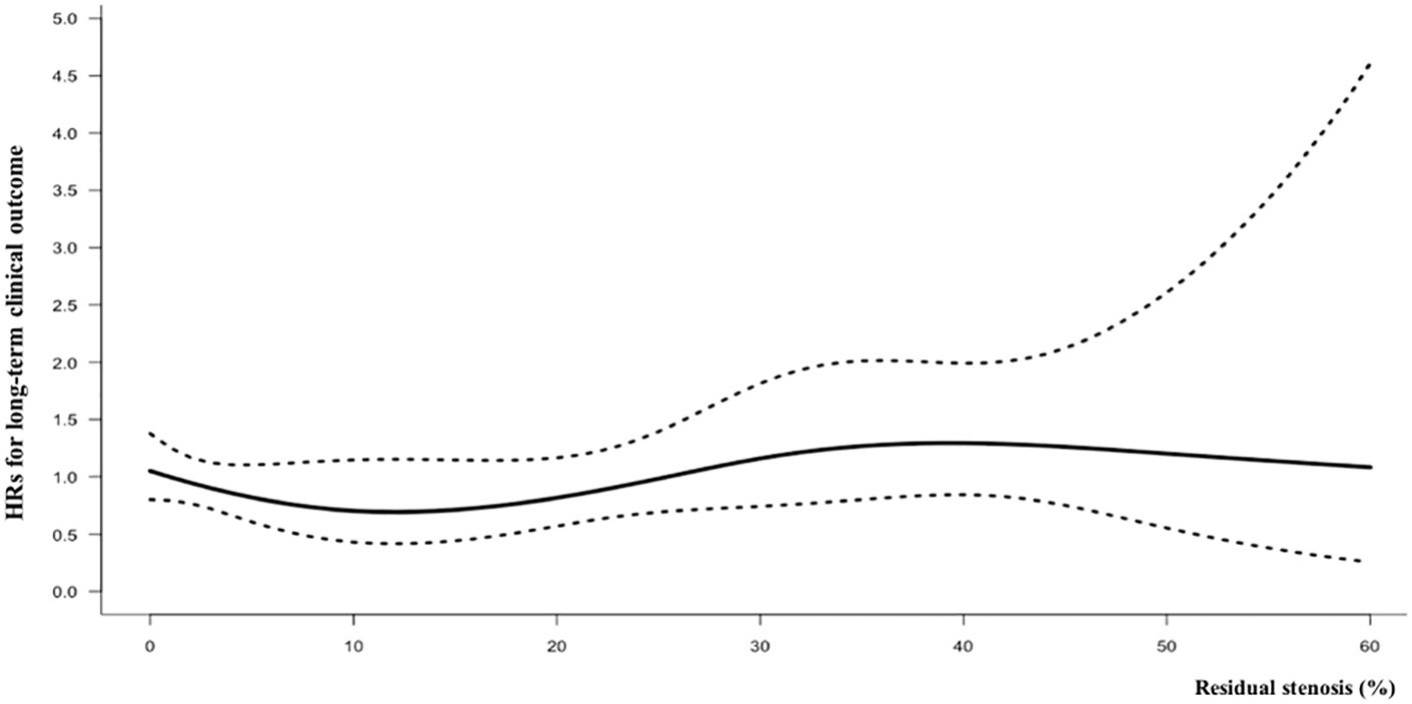
The adjusted hazard ratios of residual stenosis for clinical outcome. The solid line indicates the hazard ratio, and the dotted line indicates the 95% confidence interval. HR, hazard ratio.

The Cox proportional hazards model with restricted cubic spline smoothing method indicated that residual stenosis was not fitted to a non-linear trend with restenosis (P for non-linearity = 0.48) or clinical outcome (P for non-linearity = 0.45, Fig 3).

The WLW model indicated that residual stenosis was not independently associated with increased global hazard for restenosis or clinical outcome (adjusted hazard ratio, 1.011; 95% confidence interval, 0.997–1.025, Table 3). In a separate estimation, residual stenosis increased the hazard ratio for restenosis (1.041; 95% confidence interval, 1.012–1.072) but not clinical outcome (1.004; 0.988–1.020).

**Table 3:** The results of Wei-Lin-Weissfeld (WLW) model for long-term global and event-specific outcomes.

Variables	HR for both clinical and restenosis	HR for restenosis	HR for clinical outcome
Residual stenosis	1.011 (0.997–1.025)	1.041 (1.012–1.072)	1.004 (0.988–1.020)
Age	1.008 (0.985–1.032)	0.980 (0.920–1.044)	1.016 (0.991–1.042)
Basal stenosis	1.002 (0.983–1.022)	1.030 (0.978–1.085)	0.998 (0.978–1.018)
Hypertension	0.847 (0.558–1.601)	0.573 (0.195–1.690)	1.040 (0.550–1.969)
Statin use	0.611 (0.389–0.960)	0.498 (0.173–1.436)	0. 642 (0.387–1.066)
Symptomatic internal carotid artery	1.162 (0.753–1.793)	0.747 (0.258–2.161)	1.261 (0.793–2.060)

Values were adjusted hazard ratio (95% confidence interval) obtained from Wei-Lin-Weissfeld (WLW) models. HR was abbreviated for hazard ratio. The model fit of global model was R^2^ = 0.012 and likelihood ratio test (LR test) = 9.71 (P = 0.1). The model fit of each-event model was R^2^ = 0.03, and LR test = 12.51 (P = 0.05) for restenosis and R^2^ = 0.018, and LR test = 7.47 (P = 0.3.)

At sensitivity analysis using the Cox proportional hazard models, residual stenosis was not associated with composite of restenosis and clinical outcome (1.013; 0.999–1.027, S2 Table).

## Discussion

This study demonstrated that residual stenosis after carotid artery stenting can be helpful to predict periprocedural outcome and long-term restenosis.

An inverse relationship was observed between the degree of residual stenosis and the periprocedural outcome. That relationship appeared to be consistent across various periprocedural outcomes, especially those within 24 hours (Table 2). This finding supports the hypothesis that higher residual stenosis might contribute to decreased plaque disruption with distal embolization and hemodynamic instability.[30–32]

To analyze the long-term effect, this study used the WLW model to investigate the effect of residual stenosis for restenosis and clinical outcome. This model is used to estimate the hazards for multiple events of different types.[33] In the estimation of global effects, residual stenosis did not significantly increase the hazard for restenosis or clinical outcome. However, event-specific models indicated that residual stenosis was significantly associated with the occurrence of restenosis but not clinical outcome.

The significant association between residual stenosis and restenosis is a major concern. However, the rate of restenosis in the higher residual stenosis group (7.7%) appear to be within an acceptable range. In previous pivotal studies, the restenosis rates after CAS were 6.0% at 2 years in the Carotid Revascularization Endarterectomy versus Stenting Trial and 10.8% at 5 years in the International Carotid Stenting Study (ICSS).[2,34] In observational study, restenosis was also reported in the 8.6% for about 2 years mean follow up. [35]

Previous studies recommended early (~2 years) and frequent monitoring for restenosis and reported several risk factors, such as female sex, diabetes, and hypertension.[22,34] Our results confirm the high incidence of restenosis in the early years after CAS (Fig 3) and additionally suggest that residual stenosis may be helpful to identify patients at high risk of restenosis. Because the most of restenosis were asymptomatic, especially in mild to moderate restenosis, monitoring the recurrent carotid stenosis and prompt management would be acceptable option. [35]

Residual stenosis did not affect the long-term clinical outcomes. It is possible that the degree of residual stenosis after CAS might exert similar effects on prognosis compared to mild carotid artery stenosis, since both showed similar hemodynamic condition and burden of atheroma.[16,21]

Among the baseline characteristics, statin use decreased about 40% of the long-term risks of restenosis or clinical outcome. Biologically, pleiotropic effect of statin would exert beneficial roles. [36,37] As the recent study showed that statin use before CAS had effect of reducing the cardiovascular events, our study suggested the medical statin therapy for CAS.

This study has several limitations. First, the retrospective observational study design carries a risk of selection bias. Second, the impact of very high degrees of residual stenosis could not be assessed due to the small number of subjects with residual stenosis >50%. Third, it was unfortunate to conduct the delicate analysis including the information about the antithrombotics and statin during whole follow up period. Finally, information about procedural variables, such as types of stent and post-ballooning procedures were not assessed. Because residual stenosis is regarded as the integrated result of the procedure, the addition of those variables carried the risk of multicollinearity or over-fitting.[38]

In summary, this study demonstrated that residual stenosis could be useful to predict periprocedural outcomes. Over long-term follow-up, higher residual stenosis appears to be safe in clinically, however, it had to be cautious about restenosis.

## Supporting information

S1 Fig Histogram of the degree of residual stenosis after carotid artery stenting. (TIFF)

S1 Table Multiple logistic regression model for periprocedural outcome. (DOCX)

S2 Table The results of Cox proportional hazard models for long-term global and event-specific outcomes. (DOCX)
