# Supplementary figures and images for: Residual stenosis after carotid artery stenting: Effect on periprocedural and long-term outcomes
Source: PLoS One. 2019 Sep 9;14(9):e0216592. doi: 10.1371/journal.pone.0216592 (PMC6733450; doi:10.1371/journal.pone.0216592)

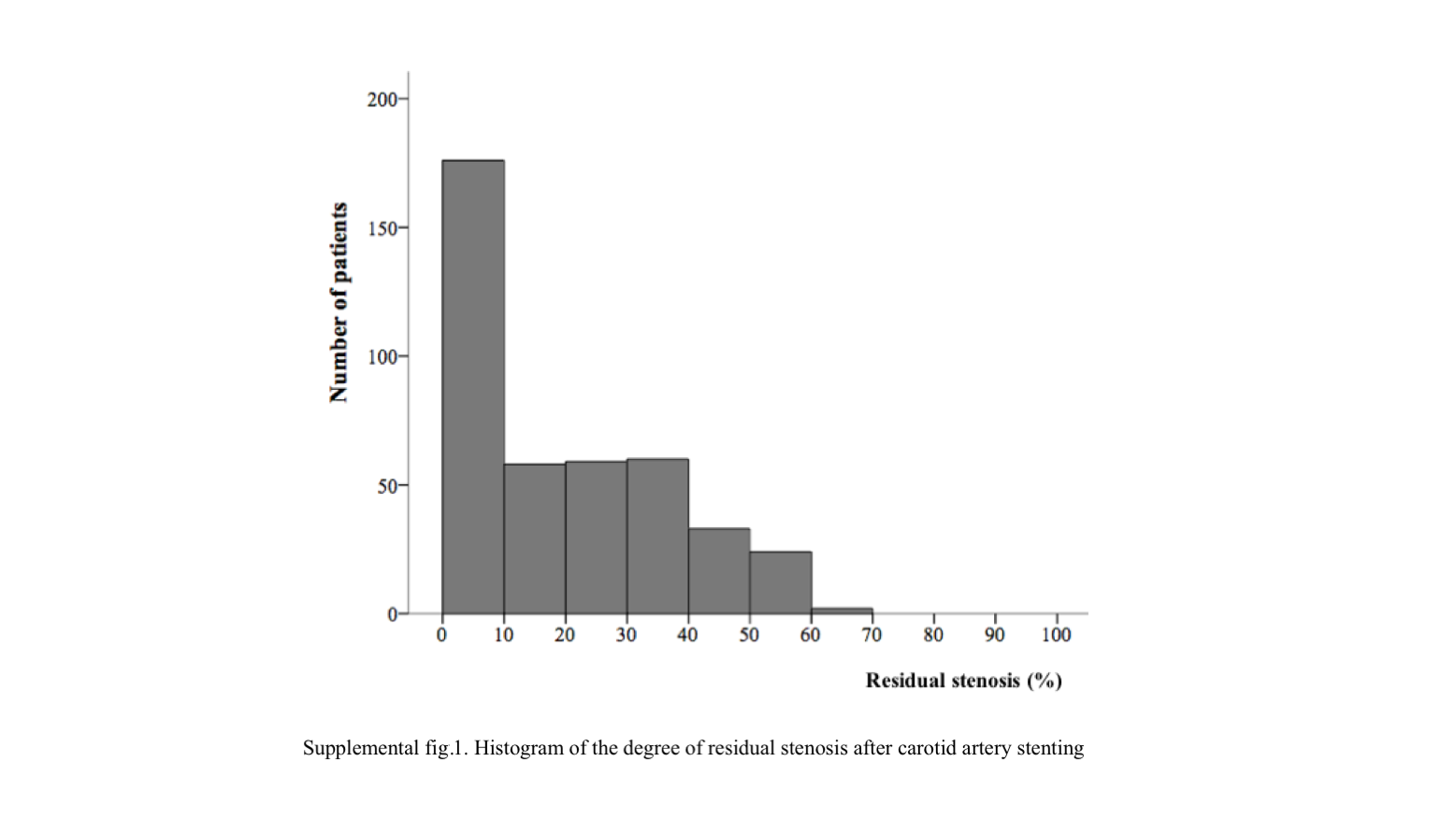

Supplement: S1 Fig — (TIFF) [file pone.0216592.s001.tiff]
