# Supplementary material for: Residual stenosis after carotid artery stenting: Effect on periprocedural and long-term outcomes
Source: PLoS One. 2019 Sep 9;14(9):e0216592. doi: 10.1371/journal.pone.0216592 (PMC6733450; doi:10.1371/journal.pone.0216592)
Supplement: S1 Table — (DOCX) [file pone.0216592.s002.docx]

**S1 Table**. Multiple logistic regression model for periprocedural outcome

| Variables | Adjusted OR (95% CI) |
| --- | --- |
| Residual stenosis | 0.983 (0.9654–0.999) |
| Age | 0.995 (0.965–1.022) |
| Hypertension | 1.370 (0.759–2.585) |
| Baseline stenosis | 0.999 (0.977–1.022) |
| Symptomaticity | 1.008 (0.603–1.696) |
| Statin use | 0.613 (0.365–1.023) |

OR, odds ratio; CI, confidence interval.

**S2 Table**. The results of Cox proportional hazard models for long-term global and event-specific outcomes.

| Variables | HR for both clinical and restenosis | HR for restenosis | HR for clinical outcome |
| --- | --- | --- | --- |
| Residual stenosis | 1.013 (0.999 – 1.027) | 1.041 (1.013 – 1.071) | 1.004 (0.989 – 1.019) |
| Age | 1.010 (0.988 – 1.032) | 0.980 (0.939 – 1.022) | 1.016 (0.992 – 1.041) |
| Basal stenosis | 1.003 (0.984 – 1.023) | 1.030 (0.977 – 1.086) | 0.998 (0.977 – 1.019) |
| Hypertension | 0.880 (0.532 – 1.457) | 0.573 (0.189 – 1.741) | 1.040 (0.592 – 1.828) |
| Statin use | 0.585 (0.379 – 0.903) | 0.498 (0.182 – 1.363) | 0.642 (0.400 – 1.032) |
| Symptomatic internal carotid artery | 1.092 (0.708 – 1.686) | 0.747 (0.272 – 2.055) | 1.261 (0.783 – 2.032) |

Values were adjusted hazard ratio (95% confidence interval) obtained from Cox proportional hazard models. HR was abbreviated for hazard ratio. The model fit of global model was R^2^ = 0.026 and likelihood ratio test (LR test) = 10.99 (P = 0.09). The model fit of each-event model was R^2^ = 0.03, and LR test =12.51 (P=0.05) for restenosis and R^2^ = 0.018, and LR test =7.47 (P=0.3.)
